# Mutations in Biosynthetic Enzymes for the Protein Linker Region of Chondroitin/Dermatan/Heparan Sulfate Cause Skeletal and Skin Dysplasias

**DOI:** 10.1155/2015/861752

**Published:** 2015-10-25

**Authors:** Shuji Mizumoto, Shuhei Yamada, Kazuyuki Sugahara

**Affiliations:** ^1^Department of Pathobiochemistry, Faculty of Pharmacy, Meijo University, 150 Yagotoyama, Tempaku-ku, Nagoya 468-8503, Japan; ^2^Laboratory of Proteoglycan Signaling and Therapeutics, Graduate School of Life Science, Hokkaido University, Sapporo 001-0021, Japan

## Abstract

Glycosaminoglycans, including chondroitin, dermatan, and heparan sulfate, have various roles in a wide range of biological events such as cell signaling, cell proliferation, tissue morphogenesis, and interactions with various growth factors. Their polysaccharides covalently attach to the serine residues on specific core proteins through the common linker region tetrasaccharide, -xylose-galactose-galactose-glucuronic acid, which is produced through the stepwise addition of respective monosaccharides by four distinct glycosyltransferases. Mutations in the human genes encoding the glycosyltransferases responsible for the biosynthesis of the linker region tetrasaccharide cause a number of genetic disorders, called glycosaminoglycan linkeropathies, including Desbuquois dysplasia type 2, spondyloepimetaphyseal dysplasia, Ehlers-Danlos syndrome, and Larsen syndrome. This review focused on recent studies on genetic diseases caused by defects in the biosynthesis of the common linker region tetrasaccharide.

## 1. Introduction

**Figure 1:**
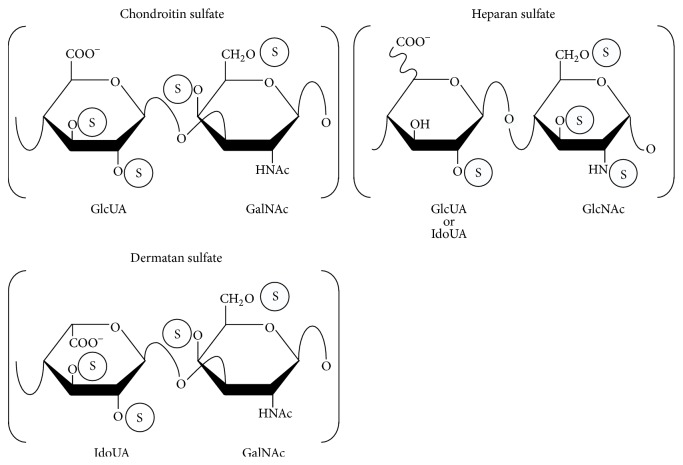
Typical repeating disaccharide units in CS, DS, and HS and their potential sulfation sites. The CS backbone consists of GlcUA and GalNAc, whereas DS is a stereoisomer of CS including IdoUA instead of GlcUA. The HS backbone consists of uronic acid and GlcNAc residues with varying proportions of IdoUA. These sugar moieties may be esterified by sulfate at various positions as indicated by the circled “S.”

Chondroitin, dermatan, and heparan sulfate (CS, DS, and HS), classified as glycosaminoglycans (GAGs), are covalently attached to specific core proteins that form proteoglycans (PGs), which are ubiquitously distributed in extracellular matrices and on cell surfaces [1]. The classification of these polysaccharides has been based on their structural units: namely, the backbone of CS is composed of repeating disaccharide units of* N*-acetyl-d-galactosamine (GalNAc) and d-glucuronic acid (GlcUA) (Figure 1), while DS consists of GalNAc and l-iduronic acid (IdoUA) instead of GlcUA (Figure 1). Both chains frequently exist as CS-DS hybrid chains in mammalian cells and tissues [2]. The backbone of HS consists of* N*-acetyl-d-glucosamine (GlcNAc) and GlcUA or IdoUA (Figure 1). These polysaccharides are modified by sulfation at various hydroxy groups and by the epimerization of GlcUA and IdoUA residues in the growing oligo- and/or polysaccharides [3–5]. These modifications provide structural diversity, thereby affecting a wide range of biological functions including cell proliferation, tissue morphogenesis, viral infections, tumor metastasis, and interactions with morphogens, cytokines, and growth factors [6–11].

The major component in cartilage is generally CS-PGs. Not only CS-PGs but also HS- and DS-PGs are expressed during bone development and regulate the maturation of chondrocyte [12]. CS side chains regulate bone morphogenetic protein and transforming growth factor-*β* signaling in the cartilage growth plate and chondrocyte columns [13]. HS or HS-PGs are known to be essential for Indian hedgehog signaling on cell surface by presenting to the receptor, Patched, in the growth plate [14]. DS-PGs, decorin and biglycan, play specific roles in all phases of bone formation including cell proliferation, matrix deposition, remodeling, and mineral deposition [15]. These observations suggest that defect in the biosynthesis of GAGs and core proteins of PGs might lead to disturbance of the skeletal development.

Genetic bone and skin disorders that are caused by mutations in the glycosyltransferases responsible for the biosynthesis of CS, DS, and HS have recently been identified [6, 8]. This review focused on recent advances in the study of cartilage and connective tissue disorders caused by disturbances in the biosynthesis of the common linker region tetrasaccharide in CS, DS, and HS chains, which have been categorized as GAG linkeropathies.

## 2. Biosynthesis of CS, DS, and HS Chains

**Figure 2:**
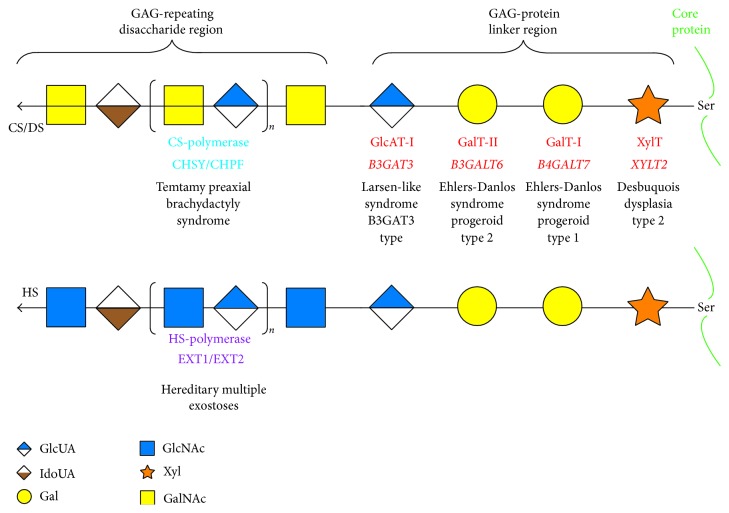
Biosynthetic assemblies of GAG-linker and GAG-disaccharide regions by various glycosyltransferases. All glycosyltransferases require a corresponding UDP-sugar, such as UDP-Xyl, UDP-Gal, UDP-GlcUA, UDP-GalNAc, and UDP-GlcNAc, as a donor substrate. After specific core proteins are synthesized, the synthesis of the common GAG-protein linker region, GlcUA*β*1-3Gal*β*1-3Gal*β*1-4Xyl*β*1-, is initiated by XylT, which transfers a Xyl residue from UDP-Xyl to the specific serine (Ser) residue(s). The linker tetrasaccharide is subsequently produced by GalT-I, GalT-II, and GlcAT-I. These four enzymes are common to the biosynthesis of CS, DS, and HS. After the formation of the linker region, CS- and HS-polymerases assemble the chondroitin and heparan backbones, respectively. Each enzyme, its coding gene, and the corresponding inheritable disorder are aligned under the respective sugar symbols from the top of each line. CHSY, CHPF, and EXT represent chondroitin synthase, chondroitin polymerizing factor, and exostosin, respectively.

The repeating disaccharide regions of CS, DS, and HS chains are attached to serine residues in core proteins through the common GAG-protein linker region tetrasaccharide, -*O*-xylose-galactose-galactose-GlcUA- (-*O*-Xyl-Gal-Gal-GlcUA-) (Figure 2) [1]. *β*-Xylosyltransferase (XylT) catalyzes the transfer of a Xyl residue from uridine diphosphate-xylose (UDP-Xyl) to specific serine residues in the newly synthesized core proteins of PGs in the endoplasmic reticulum and* cis*-Golgi compartments, which initiates the biosynthesis of CS, DS, and HS chains (Figure 2 and Table 1) [16, 17]. Two Gal residues are added to serine-*O*-Xyl in the core proteins from UDP-Gal by *β*1,4-galactosyltransferase-I (GalT-I) and *β*1,3-galactosyltransferase-II (GalT-II), which are encoded by* B4GALT7* and* B3GALT6*, respectively [18–20]. *β*1,3-Glucuronosyltransferase-I (GlcAT-I) is encoded by* B3GAT3* and then transfers a GlcUA residue from UDP-GlcUA to serine-*O*-Xyl-Gal-Gal (Figure 2 and Table 1) [21].

The subsequent construction of the backbone of the repeating disaccharide region in CS chains [-4GlcUA*β*1-3GalNAc*β*1-]_*n*_, is archived by six chondroitin synthase family members (Figure 2) [8, 9]. The formation of the backbone of the repeating disaccharide region of DS, -4IdoUA*α*1–3GalNAc*β*1-, is achieved by DS epimerase, which converts GlcUA into IdoUA by epimerizing the C5 position of GlcUA residues during or after the construction of a chondroitin backbone [4]. On the other hand, exostosin (EXT) family members, including EXT1 and EXT2 as well as EXTL1, EXTL2, and EXTL3, have been shown to catalyze the formation of the HS-backbone, [-4GlcUA*β*1-4GlcNAc*α*1-]_*n*_ [8, 35] (Figure 2).

Thereafter, these polymer chains are maturated by sulfated modifications with various sulfotransferases, which transfer the sulfate group from a sulfate donor, 3′-phosphoadenosine 5′-phosphosulfate, to the corresponding hydroxyl groups at each sugar residue of the backbone, and by epimerization of the GlcUA residue with C5-epimerases [3–5, 8, 9, 35].

## 3. GAG Linkeropathy

### 3.1. XYLT1 and XYLT2

Mutations in* XYLT1* cause an autosomal recessive syndrome that is characterized by skeletal malformations such as a short stature and femoral neck, thickened ribs, plump long bones, characteristic facial features, and intellectual disabilities [22]. A homozygous mutation in* XYLT1* was shown to result in the replacement of the amino acid, p.Arg481Trp, in the presumed catalytic domain, and the immature forms of decorin-PG without a DS side chain from the fibroblasts of the patient [22]. Furthermore, normal XYLT1 was found to be predominantly distributed in the Golgi apparatus of fibroblasts in a healthy control, whereas mutant XYLT1 was diffusely localized in the cytoplasm and partially in the Golgi in the fibroblasts of the patient [22].

Five distinct* XYLT1* mutations have been identified to date, including a missense substitution (p.Arg598Cys), nonsense mutation (p.Arg147X), truncated form mutation (p.Pro93AlafsX69), and two splice site mutations, resulting in Desbuquois dysplasia type 2, which presents severe clinical manifestations such as a short stature, joint laxity, and advanced carpal ossification [23]. In addition, the biosynthesis of high-molecular-weight CS-PGs, but not HS-PGs, was less in affected cells with these mutations in* XYLT1* than in healthy controls [23], suggesting that these manifestations may be caused by reductions in CS side chains, but not HS. These findings imply that XYLT1 mainly acts on serine residues in the core proteins of CS-PG, but not HS-PG, and that the functions of XYLT1 cannot be compensated by XYLT2.

Frameshift mutations in* XYLT2* cause an autosomal recessive syndrome that is characterized by osteoporosis, cataracts, sensorineural hearing loss, atrial septal defect, and learning difficulties similar to spondyloocular syndrome [24]. Two distinct mutations in* XYLT2* have been identified to date, including a homozygous frameshift duplication (p.Val232Glyfs^*∗*^54) and deletion (p.Ala174Profs^*∗*^35) [24]. XylT activity in serum was clearly less in affected individuals than in age-matched controls. Furthermore, fibroblasts from affected individuals were found to produce lower amount of CS and HS than those of controls [24]. These findings suggest that XYLT2 is also involved in PG assembly and is critical for normal development.

### 3.2. *B4GALT7* (GalT-I)

Mutations in* B4GALT7*, which encodes GalT-I, cause Ehlers-Danlos syndrome progeroid type 1, a disease that is characterized by an aged appearance, hypermobile joints, loose skin, craniofacial dysmorphism, a short stature, developmental delays, generalized osteopenia, and defective wound healing [25–27]. Ehlers-Danlos syndrome is a heterogeneous group of heritable connective tissue disorders characterized by joint and skin laxity as well as tissue fragility [36]. Six major categories (classical, hypermobility, vascular, kyphoscoliosis, arthrochalasia, and dermatosparaxis types) and several minor categories (progeroid, musculocontractural, cardiac valvular, periventricular nodular heterotopia, and spondylocheirodysplastic types) have been identified to date [36]. The mutants GalT-I, p.Arg270Cys, p.Ala186Asp, p.Leu206Pro, and p.Arg270Cys, exhibited lower enzymatic activities than the wild-type [27, 37–40]. Furthermore, shorter CS and HS side chains on PGs as well as the partial lack of DS side chains on decorin and biglycan core proteins have been detected in cultured cells from these patients [27, 37–40].

A recent study reported that a homozygous mutation in* B4GALT7* (p.Arg270Cys) caused a variant of Larsen syndrome in Reunion Island in the southern Indian Ocean and is characterized by multiple dislocations, dwarfism, distinctive facial features, and hyperlaxity [28]. The symptoms of Larsen syndrome are congenital large-joint dislocations and characteristic craniofacial abnormalities including dislocations of the hip, elbow, and knee and foot deformities [41]. Therefore, Ehlers-Danlos syndrome (progeroid type 1) and Larsen syndrome (in Reunion Island) may share clinical spectra including joint dislocations.

### 3.3. *B3GALT6* (GalT-II)

Compound heterozygous mutations in* B3GALT6* encoding GalT-II cause Ehlers-Danlos syndrome progeroid type 2 [29, 30]. Three frameshift and two missense mutations have been identified in three patients [29]. Recombinant GalT-II mutant (p.Ser309Thr) exhibited significantly lower GalT-II activity than that of the wild-type enzyme [29]. Moreover, mutations in* B3GALT6* have also been shown to cause an autosomal-recessive disorder, spondyloepimetaphyseal dysplasia with joint laxity type 1, which is characterized by kyphoscoliosis, clubfeet, hip dislocation, elbow contracture, platyspondyly, and craniofacial dysmorphisms including a small mandible with a cleft palate, prominent eyes, and a long upper lip [29–31]. Skeletal and connective manifestations in Ehlers-Danlos syndrome progeroid type 2 and spondyloepimetaphyseal dysplasia with joint laxity type 1 largely overlap; however, these patients share no common mutations in* B3GALT6* [29]. The recombinant enzymes, p.Ser65Gly-, p.Pro67Leu-, p.Asp156Asn-, p.Arg232Cys-, and p.Cys300Ser-GalT-II, were found to exhibit significantly lower galactosyltransferase activities than that of wild-type GalT-II [29]. Although wild-type GalT-II is expressed in the Golgi, the mutant enzyme (p.Met1?), which affects the initiation codon, c.1A>G, is located in the cytoplasm and nucleus [29], suggesting that mislocalization of the mutant protein may cause GalT-II dysfunctions.

Furthermore, Malfait et al. identified three missense mutations and one frameshift mutation in* B3GALT6* in patients exhibiting various symptoms similar to those of Ehlers-Danlos syndrome and spondyloepimetaphyseal dysplasia with joint hyperlaxity [30]. Cultured fibroblasts from the affected individuals synthesized markedly less GAG and the DS side chain on decorin was absent. These findings indicated that not only the enzymatic activity, but also cellular localization of the mutant proteins affects the biosynthesis of CS, DS, and HS chains, leading to the abnormal development of skin, bone, and connective tissues.

### 3.4. *B3GAT3* (GlcAT-I)

A mutation (p.Arg277Gln) in the* B3GAT3* gene encoding GlcAT-I has been shown to cause Larsen-like syndrome [32, 33]. These patients predominantly have elbow dislocations with a bicuspid aortic valve in the heart as well as the characteristic manifestations of Larsen syndrome [32]. GlcA-transferase activity was markedly lower in the recombinant GlcAT-I mutant (p.Arg277Gln) and fibroblasts of these patients than in wild-type and healthy controls, respectively [32]. Moreover, fibroblasts from patients lacked the CS, DS, and HS side chains on PGs found in control cells.

A mutation in GlcAT-I (p.Pro140Leu) was recently identified from Nias island, Indonesia, in eight patients with a short stature as well as bone dysplasia including scoliosis, midface hypoplasia, dislocation of joints, broad ends of fingers and toes, and foot deformities [34]. The transferase activity of the recombinant GlcAT-I mutant (p.Pro140Leu) appeared to be lower than the wild-type [34]. Furthermore, the amounts of CS, DS, and HS from the lymphoblastoid cells of these patients were markedly lower than those of healthy controls [34]. These findings suggested that this mutation in* B3GAT3* (GlcAT-I) affected the biosynthesis of CS, DS, and HS side chains on PGs, leading to abnormal bone development.

## 4. Conclusions

Recent advances in genetic and glycobiological studies on connective tissue disorders have clarified the biological significance of GAG side chains and their linker region tetrasaccharides of PGs [6, 8]; however, the underlying pathogenic mechanisms remain unclear. Faulty biosynthesis of GAGs and PGs might affect assembly of matrix proteins and cell signaling during skeletal formation in GAG linkeropathies.

Various manifestations of GAG linkeropathies are clearly caused by mutations in the four glycosyltransferases responsible for the biosynthesis of the three kinds of polysaccharide chains, CS, DS, and HS, which are constructed on the core proteins of PGs. However, the clinical symptoms of GAG linkeropathies do not always agree among the different mutations of a glycosyltransferase nor the four distinct glycosyltransferases, XylT1, GalT-I, GalT-II, and GlcAT-I. These varieties of phenotypes may partially be due to distinct residual enzymatic activities, the cellular mislocalizations of mutant proteins, or partial compensation of the loss of function in each glycosyltransferase by other homologues, which may affect the quality of the GAGs yielded, that is, different lengths, numbers, and sulfation patterns of GAGs in the affected individuals.

Further comprehensive studies on the molecular pathogeneses of GAG linkeropathies are required for the development of design of new therapeutics for these diseases.

## Figures and Tables

**Table 1:** Biosynthetic enzymes of the GAG-linker region tetrasaccharide.

Enzymes (activity)	Coding genes	Chromosomal location	MIM number	Human genetic disorders (GAG linkeropathies)	Amino acid changes in disorders	References
Xylosyltransferase (XylT)	*XYLT1 *	16p12.3	608124 615777	Desbuquois dysplasia type 2, short stature syndrome	Pro93Alafs^*∗*^69; Arg147X; Arg481Trp; Arg598Cys; two mutations in splice site	[22, 23]
	*XYLT2 *	17q21.33	608125	Spondyloocular syndrome with bone fragility, cataracts, and hearing defects	Ala174Profs^*∗*^35; Val232Glyfs^*∗*^54	[24]

*β*4-Galactosyltransferase-I (GalT-I)	*B4GALT7 *	5q35.2-q35.3	130070 604327	Ehlers-Danlos syndrome progeroid type 1, Larsen of Reunion Island syndrome	Ala186Asp; Leu206Pro; Arg270Cys	[25–28]

*β*3-Galactosyltransferase-II (GalT-II)	*B3GALT6 *	1p36.33	271640 615349 615291	Ehlers-Danlos syndrome progeroid type 2, spondyloepimetaphyseal dysplasia with joint laxity type 1	Met1?; Arg6Trp; Ser65Gly; Pro67Leu; Ala108Glyfs^*∗*^163; Asp118Alafs^*∗*^160; Met139Ala141del; Asp156Asn; Arg197Alafs^*∗*^81; Asp207His; Gly217Ser; Arg232Cys; Cys300Ser; Ser309Thr	[29–31]

*β*3-Glucuronyltransferase-I (GlcAT-I)	*B3GAT3 *	11q12.3	245600 606374	Larsen-like syndrome B3GAT3 type Multiple joint dislocations, a short stature, craniofacial dysmorphism, and congenital heart defects	Pro140Leu; Arg277Gln	[32–34]

## Abbreviations

*B3GALT6*: Beta-1,3-galactosyltransferase 6
*B4GALT7*: Beta-1,4-galactosyltransferase 7
*B3GAT3*: Beta-1,3-glucuronyltransferase 3
CS: Chondroitin sulfate
DS: Dermatan sulfate
GAG: Glycosaminoglycan
GalNAc: * N*-Acetyl-d-galactosamine
GlcUA: d-Glucuronic acid
HS: Heparan sulfate
IdoUA: l-Iduronic acid
PG: Proteoglycan
XYLT: Beta-xylosyltransferase.
